# An Optimized Screening Approach for the Oxazolidinone Resistance Gene *optrA* Yielded a Higher Fecal Carriage Rate among Healthy Individuals in Hangzhou, China

**DOI:** 10.1128/spectrum.02974-22

**Published:** 2022-11-15

**Authors:** Weiyi Shen, Yonglu Huang, Jiachang Cai

**Affiliations:** a Clinical Microbiology Laboratory, The Second Affiliated Hospital of Zhejiang University School of Medicine, Zhejiang University, Hangzhou, China; Pontificia Universidade Catolica do Paraná

**Keywords:** *optrA*, screening method, fecal carriage, genetic environment, linezolid resistance, screen method

## Abstract

The linezolid resistance mediated by *optrA* has exhibited an increasing trend among Gram-positive bacteria, which greatly limits the treatment options for severe bacterial infections. However, the prevalence of *optrA* was usually underestimated based on the existing screening methods. In this study, we used a traditional method and an improved method that included a high-salinity condition treatment after enrichment to screen for *optrA*-carrying bacteria from stool samples from 1,018 healthy donors in Hangzhou, China. The fecal carriage rate of *optrA*-carrying bacteria was 19.25% when screened by the improved method (196/1,018), which was much higher than that of the traditional method at 5.89% (60/1,018). Enterococci were the majority of the *optrA*-positive isolates, while five nonenterococcal isolates were also obtained, including two Streptococcus gallolyticus, one Vagococcus lutrae, one Lactococcus garvieae, and one Lactococcus formosensis isolate. Whole-genome sequencing analysis identified four novel OptrA variants, IDKKGPM, IDKKGP, KLDK, and EYDDI, in these isolates, whose *optrA-*flanking regions with a *fexA* gene downstream were bounded by different insertion sequences. In conclusion, our optimized method displayed high sensitivity in the detection of *optrA*-positive bacteria in fecal samples and revealed a high carriage rate in a healthy population. Although enterococci are dominant, multiple *optrA*-carrying Gram-positive bacteria were also found.

**IMPORTANCE** This study represented an optimized screening approach for the *optrA* gene, which is an important mechanism of antimicrobial resistance to linezolid as a last resort for the treatment of infections caused by multiresistant Gram-positive bacteria. We revealed a high fecal carriage rate of the *optrA* gene among adults by this method and reported the first identification of *optrA* in Lactococcus formosensis as well as the identification of this gene in Vagococcus lutrae and of the *poxtA* gene in Ligilactobacillus salivarius of human origin, suggesting the wide spread of the *optrA* gene in the Gram-positive bacterial community.

## INTRODUCTION

Linezolid, the first member of the oxazolidinone class of antimicrobial agents, was approved in 2000 for clinical use as an effective alternative for the treatment of infections caused by multiresistant Gram-positive bacteria (1). By binding to the large subunit of the bacterial ribosome via interaction with 23S rRNA to inhibit bacterial protein synthesis, linezolid demonstrates excellent activity against Gram-positive pathogens *in vitro* (2). However, the emergence of linezolid resistance mediated by the *optrA* gene has been reported worldwide (3). Additionally, several transferable resistance determinants were also proven to confer resistance to this antibiotic, including point mutations in domain V of the 23S rRNA (4) and ribosomal proteins (5), along with the transferable multiresistant genes *cfr* and *poxtA* (6).

The *optrA* gene encodes an ATP-binding cassette F (ABC-F) protein, resulting in resistance to phenicols and oxazolidinones by executing a ribosomal protection function (3, 7). Initially described on a plasmid of a clinical Enterococcus faecalis isolate in China in 2015, the *optrA* gene has been shown to be broadly disseminated among *Enterococcus* spp. both of human origin and in environmental samples from the Asia-Pacific region, Europe, the Americas, and Africa (7–13). According to the surveillance data provided by the ZAAPS and SENTRY programs (5, 14), the *optrA* gene has now become more prevalent as the common mechanism in linezolid-resistant E. faecalis and the sole oxazolidinone resistance mechanism than the alterations in 23S rRNA, which were reported to be the main cause for enterococcal resistance previously (15). Moreover, this gene was also observed in other Gram-positive bacteria including Staphylococcus aureus, Streptococcus agalactiae, Streptococcus gallolyticus, and Lactococcus garvieae in clinical settings (16, 17), while other Staphylococcus spp., Streptococcus suis, Streptococcus parasuis, Clostridium perfringens, Listeria monocytogenes, and Vagococcus lutrae (16, 18, 19) were from animals or environmental samples exclusively.

**FIG 1:**
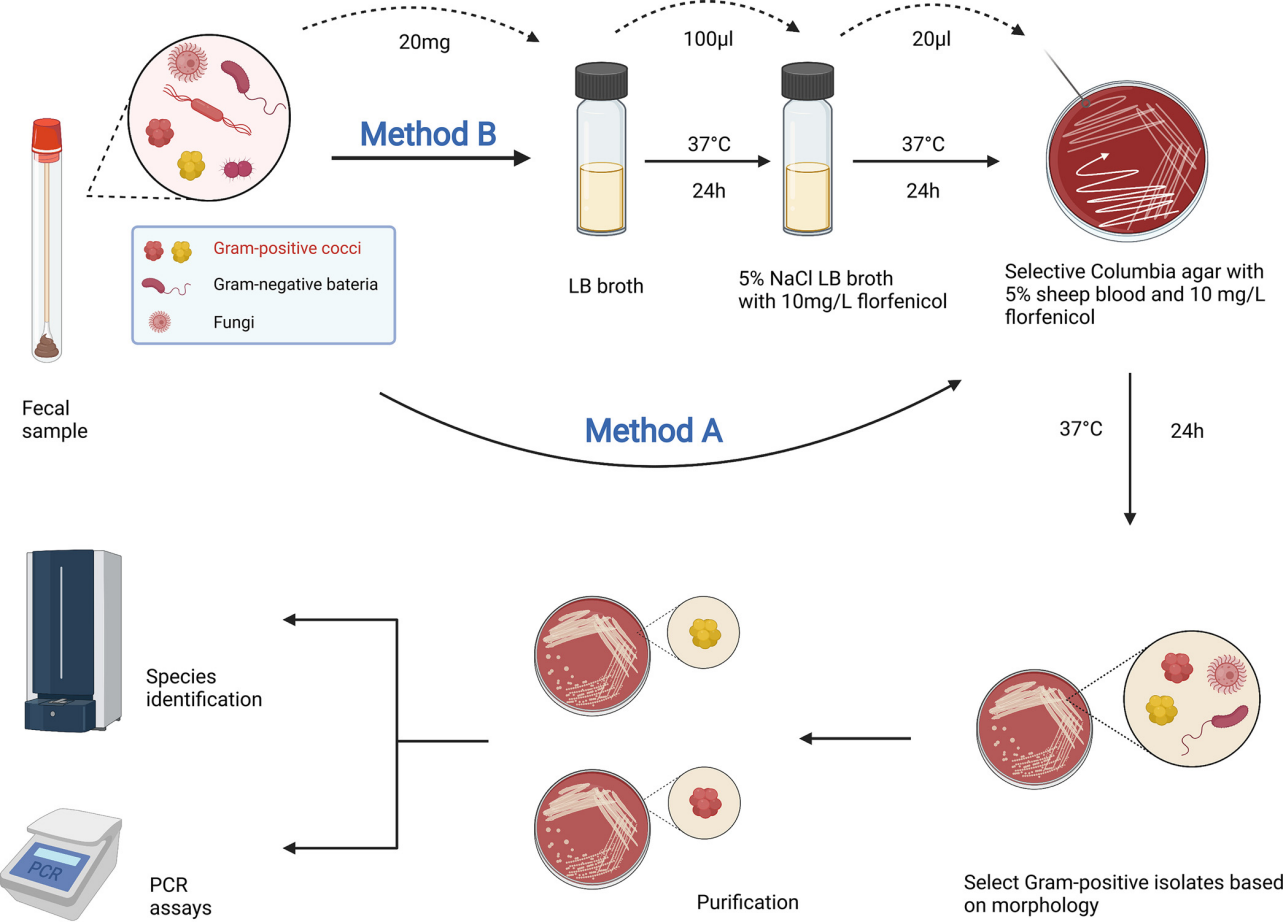
The traditional method (method A) and the improved enrichment approach (method B) to screen for *optrA*-carrying Gram-positive bacteria.

In 2019, we reported fecal carriage rates of 3.53% for *optrA*-positive enterococci in adults (20). Considering the transferability and rapid dissemination of *optrA* as previously described (21, 22), the distribution of *optrA* was potentially underestimated, and this was also limited by the method of detection. In addition, previous studies concerning the prevalence of the *optrA* gene in clinical settings were mainly restricted to enterococci (16). Thus, to understand the comprehensive epidemiology of *optrA* and move closer to determining its real prevalence, we present an improved enrichment approach to screen for *optrA*-carrying Gram-positive cocci (Fig. 1), by which we evaluated the prevalence of these strains in the intestines of healthy individuals. Furthermore, in view of the genetic environment surrounding the *optrA* gene in enterococci, which was well characterized previously (16), we performed whole-genome sequencing (WGS) analysis for nonenterococcal isolates to investigate the genetic context of the *optrA* gene in these isolates.

## RESULTS

### Isolation and distribution of *optrA*-carrying Gram-positive isolates.

**FIG 2:**
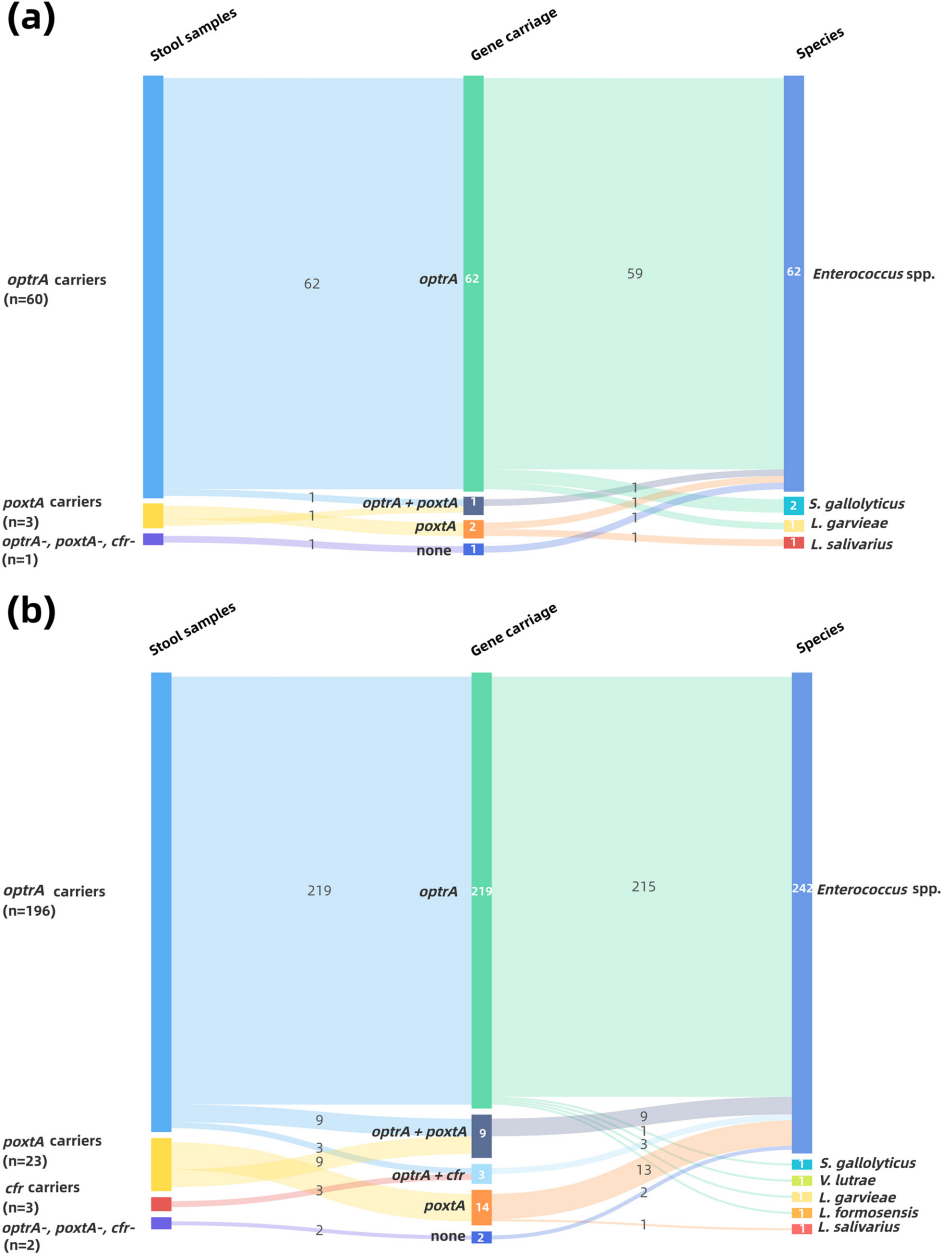
Fecal carriage rates and distribution of oxazolidinone resistance genes in isolates of collection A (a) and collection B (b).

A total of 66 florfenicol-resistant Gram-positive isolates were collected using method A (collection A; Fig. 2a), 63 of which were positive for *optrA* isolated from 60 stool samples with a carriage rate of 5.89% (60/1,018). Only 3 (carriage rate of 0.29%) isolates were positive for *poxtA*, including Enterococcus faecium A1010-1 concomitantly carrying *optrA* and *poxtA.* None of the isolates were positive for the *cfr* gene, and we also observed that E. faecium A156 possessed none of the three determinants. Among 63 *optrA*-positive isolates, enterococci were predominant and accounted for 95.24% (60/63), whereas one L. garvieae (strain A974) and two S. gallolyticus (strains A65 and A547) isolates were also included (see Table S1 in the supplemental material), the latter of which has never been reported in China previously.

For method B, 247 florfenicol-resistant Gram-positive isolates were obtained (collection B; Fig. 2b), including 231 isolates that were positive for *optrA* derived from 196 samples, which demonstrated a carriage rate of 19.25% (196/1,018) for *optrA*-carrying Gram-positive isolates in the intestines of healthy individuals. The *poxtA* gene was detected in 23 isolates with a carriage rate of 2.26%, and nine of them were positive for *optrA* concurrently. In addition, three *optrA*-carrying enterococci including one Enterococcus raffinosus (strain B495), one Enterococcus casseliflavus (strain B516), and one Enterococcus avium (strain B732) isolate demonstrated the concomitant occurrence of *cfr* (carriage rate of 0.29%). E. faecium A156, B156, and B113 were negative for all three resistance determinants. Similarly, *Enterococcus* spp. still had the highest frequency in collection B (242/247); however, except for the two genera of Gram-positive isolates included in collection A, one *poxtA*-carrying Ligilactobacillus salivarius strain B27-2 (included in collection A as A27) and one *optrA*-carrying *V. lutrae* strain B391-2 were also obtained (Table S1), both of which were reported only as porcine-origin isolates (18, 23). Furthermore, one *optrA*-positive Lactococcus formosensis (strain B827) isolate was isolated, and such strains have never been reported before.

Unlike S. gallolyticus A65 (included as strain B65 in collection B), the absence of the corresponding isolate in collection B for strain A547 was noticed, which also held true for six enterococcal isolates in collection A (strains A167, A245, A391, A592-2, A670, and A1010-2). It was tempting to speculate that a high-salinity treatment inhibited the growth of these isolates; thus, they were inoculated into fresh LB broth with 5% NaCl individually and streaked on the abovementioned selective medium to determine whether the original strains had grown. Surprisingly, except for S. gallolyticus A547, six other enterococcal isolates were derived from the selective medium after treatment, which was further confirmed by species identification and the presence of *optrA*. S. gallolyticus A547 was absent, indicating that a high-salinity environment was possibly not conducive for this isolate or at least that the isolate was not the predominant population in this environment.

### Comparison of *optrA* carriage rates among different groups.

According to the results obtained with both methods, the fecal carriage rates of *optrA* were not gender related (for method A, male/female ratio = 6.56%:5.12%, *P* = 0.331; for method B, male/female ratio = 19.67%:18.76%, *P* = 0.714). Among different age groups, the *optrA* carriage rates ranged from 14.28% to 30.26% (*P* = 0.067) when using method B but varied in a broader range of 3.57% to 14.47% when using method A; additionally, a significantly higher rate was noticed in the fecal samples of the 70- to 79-year-old age group (*P* = 0.025). However, the fecal carriage rates of *optrA*-positive enterococci among different age groups revealed no statistical significance regardless of the method used (*P* = 0.050 and *P* = 0.055 for method A and method B, respectively).

### Antimicrobial susceptibility results.

As shown in Table S1, all of the *optrA*-positive isolates were either intermediate or resistant to chloramphenicol, while two *L. salivarius* and one Enterococcus hirae isolate exclusively carrying the *poxtA* gene remained susceptible to this antibiotic with MIC values of 8 mg/L. The linezolid resistance rate of E. faecalis (70.37% for collection A and 82.59% for collection B) was approximately twice that of E. faecium (37.5% for collection A and 39.66% for collection B). Ten *optrA*-positive isolates with an additional carriage of *poxtA* were interpreted as intermediate or borderline resistant to linezolid with MIC values of 4 to 8 mg/L. For the isolates carrying the *cfr* and *optrA* genes, their linezolid MICs were only 2 to 4 mg/L. All of the E. faecalis isolates remained susceptible to penicillin G (MIC of <8 mg/L), which was in line with our previous study (20). A similar resistance rate for ciprofloxacin was shared by collection A and collection B at 43.94% and 43.72%, respectively. No vancomycin-resistant (except Enterococcus gallinarum, *E. casseliflavus*, and *L. salivarius*, which are intrinsically vancomycin resistant) isolates were observed. Both collections displayed high rates of resistance to erythromycin and tetracycline at approximately 80% and 91%, respectively. Notably, four enterococcal isolates in collection B (strains B252-2, B398-1, B539, and B556) showed susceptibility profiles distinguishable from those of their equivalent isolates in collection A (strains A252, A398, A539, and A556, respectively), despite being identified and classified into the same species, indicating the presence of multiple clones of the same species in one sample.

### Identification of resistance determinants and OptrA variants in nonenterococcal isolates.

Five *optrA*-positive nonenterococcal isolates including two S. gallolyticus (strains A547 and B65), one L. garvieae (strain B974), one *L. formosensis* (strain B827), and one *V. lutrae* (strain B391-2) isolate and one *poxtA*-positive *L. salivarius* (strain B27-2) isolate were subjected to WGS analysis. These isolates displayed multicarriage of resistance determinants (Table S2), and one of the phenicol resistance genes, *fexA* or *fexB*, was detected. The erythromycin resistance gene *erm*(B) was detected in S. gallolyticus A547 and B65 and *L. formosensis* B827, while strain S. gallolyticus A547 carried an additional *erm*(A) gene. Except for L. garvieae B974, a variety of *tet* genes [*tet*(L), *tet*(M), *tet*(O), *tet*(O/W/32/O), and *tet*(S)] were found, which conferred the resistance to tetracycline for these isolates with MIC values of >32 mg/L. Additionally, other resistance genes, including the ATP-binding cassette (ABC) antibiotic efflux pump gene *lsa*(E), lincosamide adenylation enzyme genes of the *lnu* family [*lnu*(A), *lnu*(B), and *lnu*(G)], aminoglycoside-inactivating enzyme genes [*aac(6′)-aph(2′')* and *ant(6)-Ia*], and multidrug efflux transmembrane transporter gene *mdt*(A), were detected in one or more isolates.

A total of four novel OptrA variants were obtained, including two resembling novel OptrA variants IDKKGPM and IDKKGP identified in S. gallolyticus A547 and B65, respectively. Both shared the alterations K3I, G40D, T112K, E290K, S411G, and T481P, while the substitution I622M additionally constituted the former. Two *Lactococcus* isolates demonstrated different OptrA variants with a novel variant EYDDI (K3E, N12Y, Y176D, G393D, and N559I) for L. garvieae B974 but the wild type for *L. formosensis* B827. Moreover, *V. lutrae* C391-2 possessed another OptrA variant, KLDK, which showed alterations T112K, S147L, Y176D, and I287K.

### Genetic environment of the *optrA* gene.

**FIG 3:**
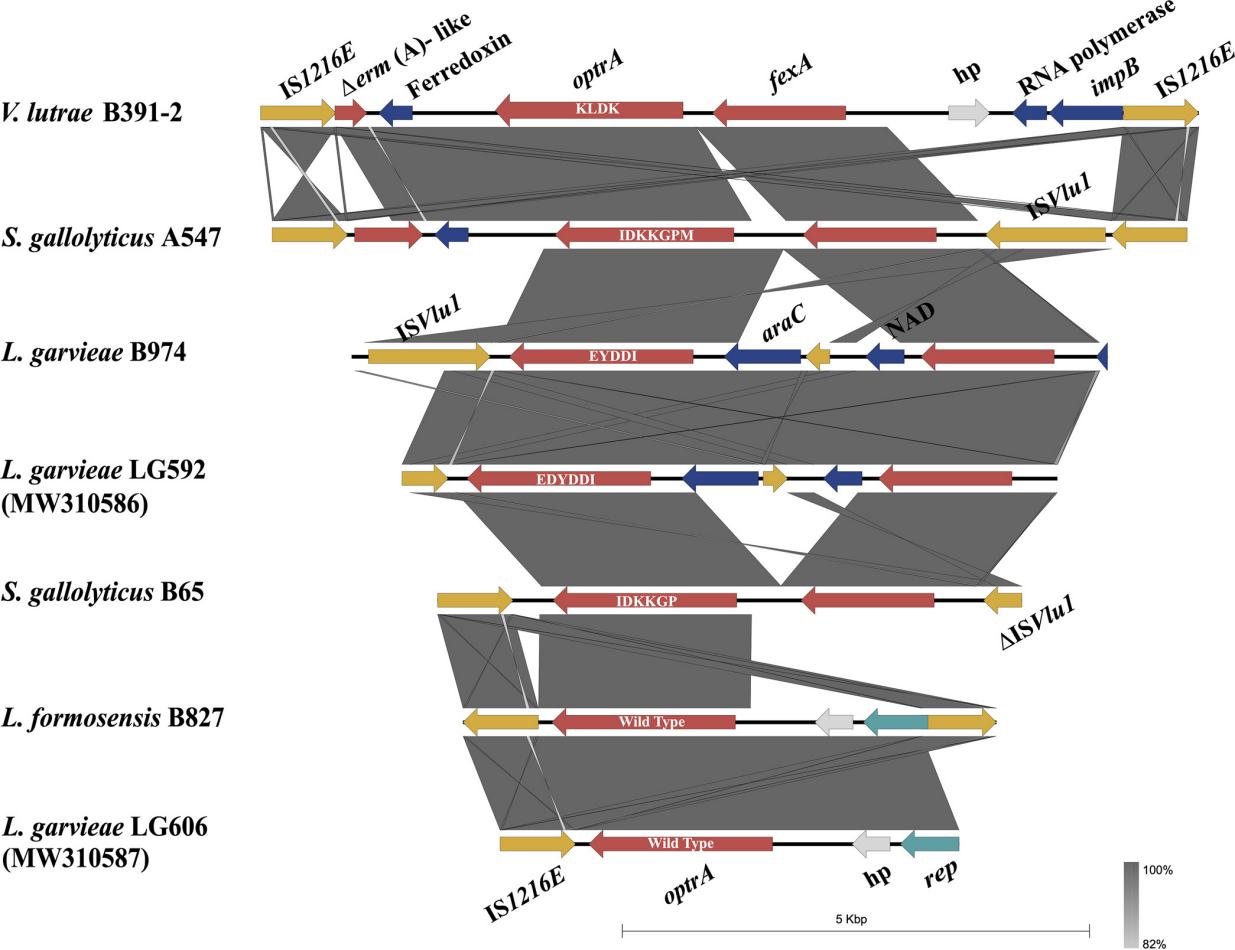
Genetic environment of five *optrA*-positive nonenterococcal isolates in this study and two known *L. garvieae* isolates (strains LG592 and LG606). The genes of different functions are labeled with different colors, and the arrows indicate the positions and directions of transcription of the different genes. The types of OptrA variants are displayed on the bar of the *optrA* gene in white font. Δ indicates a truncated gene.

As shown in Fig. 3, different insertion sequences (ISs) were found upstream and/or downstream of *optrA* in five nonenterococcal isolates. Although the occurrence of *optrA* in S. gallolyticus has been reported in Thailand (14), its genetic environment has yet to be described. Two copies of IS*1216E* elements in the opposite orientation were found to bracket the *optrA*-carrying central region of S. gallolyticus A547, which contains a truncated *erm*(A)-like gene and a ferredoxin-encoding gene upstream of the “*optrA*-*fexA*” segment. This structure was also observed in *V. lutrae* B391-2 but differed from S. gallolyticus A547 with an intact IS*VLU1* downstream, which belongs to the IS*L3* family. For S. gallolyticus B65, the “*optrA*-*fexA*” segment was directly located between IS*1216E* and IS*VLU1* oriented in the opposite direction. The three *optrA*-carrying contigs described above displayed >99% identity to a variety of corresponding sequences of enterococci according to the NCBI database. With a copy of IS*VLU1* upstream, the “*optrA*-*fexA*” segment in L. garvieae B974 was interrupted by the intergenic array of the transcriptional regulator gene *araC*, a truncated IS*VLU1*, and a putative gene encoding an uncharacterized NAD(P)H oxidoreductase. This arrangement was shared with plasmid pLG592-optrA in L. garvieae LG592 described in our previous study (17) with 99.97% identity, which additionally carried an OptrA variant, EDYDDI, similar to that of L. garvieae B974 (EYDDI). Bounded by two copies of IS*1216E* likewise, a *rep* gene was introduced downstream of *optrA* in *L. formosensis* B827, which had a sequence surrounding *optrA* that exhibited high similarity (100% identity) to a previously reported plasmid pLG606-optrA of *L. garvieae* LG606 (17), and the OptrA in both isolates was regarded as wild type.

## DISCUSSION

The linezolid resistance mediated by the *optrA* gene has shown a rapidly increasing trend worldwide, which compromises the effectiveness of the treatment of bacterial infections and represents a great threat to public health. Due to the low sensitivity of traditional screening methods, the accurate fecal carriage rate of *optrA*-positive bacteria was underestimated. This study presented an optimized enrichment approach for the detection of *optrA*-carrying bacteria, which provided a much more effective reflection of the population carriage of this determinant. Compared to the traditional method previously used, a much higher fecal carriage rate of 19.25% among healthy individuals was observed when using this improved method, suggesting the widespread occurrence of this resistance gene. Based on the traditional method (method A), two selection processes were supplemented in method B, the first of which was a high-salinity condition containing florfenicol that expanded the resistant strains and inhibited the growth of Gram-negative strains and the other of which was the selective medium with florfenicol that screened for the target isolates. Both selection processes contributed to reducing the interference of other strains that may cover the target isolates and made them easier to isolate. Thus, using method B permitted the identification of not only the isolates obtained by method A but also additional isolates that were not detected by method A, which contributed to a higher carriage rate estimated with method B.

However, this carriage rate was still possibly underestimated with several limitations. Due to the abundance of Gram-negative bacteria in the fecal samples, the target isolates were likely to be covered by these overwhelming undesired strains that were also resistant to florfenicol and the high-salinity environment, especially based on the lower quantity for Gram-positive isolates. Thus, it was unlikely that all of the *optrA*-positive target strains were observed and isolated, and this conceivably contributed to the scenario for the lack of corresponding isolates in collection B of those high-salinity-resistant enterococci we observed previously (strains A167, A245, A391, A592-2, A670, and A1010-2). In this study, two S. gallolyticus isolates demonstrated different resistance to a high-salinity condition, which resulted in the absence or presence of relevant isolation for S. gallolyticus A547 and A65, respectively, when using the optimized method. Thus, the different levels of resistance to a saline environment for some Gram-positive strains might be another influence involved in the overlooked prevalence of *optrA*.

Furthermore, we described the occurrence of *optrA* in *V. lutrae* and *poxtA* in *L. salivarius.* To the best of our knowledge, this is the first identification of these strains in samples of human origin. Four novel OptrA variants were identified in these nonenterococcal isolates, and their *optrA*-flanking regions, which resembled those in *Enterococcus* spp., were investigated, suggesting the dissemination of *optrA* among a broader group of Gram-positive members. Hence, the active surveillance of *optrA* carriers should also be conducted on nonenterococcal bacteria due to their equal role as reservoirs of this resistance mechanism.

In conclusion, we developed an optimized alternative screening method for *optrA*-positive bacteria that displayed high sensitivity in the detection of gene carriage, which provided a sounder footing for routine surveillance and further investigation of this resistance mechanism. Our results also showed the high intestinal carriage rate of the *optrA* gene among healthy individuals and its widespread distribution in Gram-positive bacterial communities. Thus, the prevalence of *optrA* must be intensively monitored to prevent and control the further dissemination of this linezolid resistance mechanism.

## MATERIALS AND METHODS

### Isolation and species identification.

From February to April 2022, a total of 1,018 nonduplicated stool samples from asymptomatic healthy individuals (549 males and 469 females) who received health examinations in a tertiary care hospital in Hangzhou, China, were collected. All the samples were processed to screen for the *optrA*-carrying isolates with the method in our previous study (method A) (20), as well as an optimized enrichment method (method B) concurrently with the following steps (Fig. 1): first, 20 mg of each stool sample was inoculated into 5 mL of Luria-Bertani (LB) broth within 4 h of collection and incubated at 37°C for 24 h; then, 100 μL of each enriched sample was transferred to a subculture of 5 mL fresh LB broth containing 5% NaCl and 10 mg/L florfenicol for another 24 h of incubation. Then, 20 μL of the mixture was streaked onto a selective medium consisting of Columbia agar base supplemented with 5% (vol/vol) sheep blood and 10 mg/L of florfenicol and kept at 37°C for 24 h. Based on the morphology, the putative target isolates were selected from the colonies that grew on the selective media and subcultured on the fresh ones for purification. The species identification was determined by matrix-assisted laser desorption ionization–time of flight mass spectrometry (MALDI-TOF MS) (Bruker Daltonik GmbH, Bremen, Germany). The nonenterococcal strains were further confirmed by comparison with reference strains using an online tool, an average nucleotide identity (ANI) calculator for certain isolates (http://enve-omics.ce.gatech.edu/ani/index). The figure for the workflows of the screening methods (Fig. 1) was created with BioRender (https://biorender.com). To compare the *optrA* carriage rates among the samples, the participants were divided into seven groups according to their age (ages 20 to 29, 30 to 39, 40 to 49, 50 to 59, 60 to 69, 70 to 79, and above 80 years). The chi-square test was used to compare the rates or proportions. A *P* value of <0.05 was considered statistically significant.

### Detection of transferable oxazolidinone resistance determinants and antimicrobial susceptibility testing.

All isolates identified as Gram-positive bacteria were screened for the carriage of oxazolidinone resistance determinants including *cfr*, *optrA*, and *poxtA* by PCR assays and Sanger sequencing. The OptrA variants were determined by comparison of the deduced amino acid sequences of the isolates with that of the original OptrA from E. faecalis E349, which was previously designated the wild type (3). The MICs of seven antimicrobial agents including linezolid, chloramphenicol, penicillin G, vancomycin, ciprofloxacin, erythromycin, and tetracycline were determined using the broth microdilution method (24) and interpreted according to the Clinical and Laboratory Standards Institute (CLSI) standard (25, 26). The interpretive criteria for susceptibility testing of *V. lutrae* are still unavailable at present. Therefore, the susceptibility breakpoints of seven antimicrobial agents for enterococci were applied for *V. lutrae* due to the close genetic relatedness of the two genera. E. faecalis ATCC 29212, Staphylococcus aureus ATCC 29213, and Streptococcus pneumoniae ATCC 49619 were employed as the quality control strains.

### WGS and genome analysis.

The genomic DNA extracted from six nonenterococcal isolates was subjected to whole-genome sequencing (WGS) using the Illumina NovaSeq 6000 platform, and the sequencing data were *de novo* assembled into contigs by SPAdes v.3.13.1 (27). Carriage of the antimicrobial resistance genes for the assembly scaffolds was determined with default settings by ResFinder 4.1 (28), available at the Center for Genomic Epidemiology (https://cge.food.dtu.dk/services/ResFinder/). BLASTN analysis was performed to compare the contigs containing the target genes with known sequences of the NCBI database. Easyfig (v2.2.2) was used to visualize the linear alignment of the genetic environment of the *optrA* gene in different isolates (29).

### Ethics approval.

This study was approved by the Ethics Committee of The Second Affiliated Hospital of Zhejiang University School of Medicine.

### Data availability.

The genomes of Ligilactobacillus salivarius B27-2, L. garvieae B974, *V. lutrae* B391-2, S. gallolyticus B65, S. gallolyticus A547, and Lactococcus formosensis B827 have been deposited in the NCBI GenBank under accession numbers JAMXTA000000000, JAMXTB000000000, JAMXTC000000000, JAMXTD000000000, JAMXTE000000000, and JAMXTL000000000, respectively. Data for carriage of oxazolidinone resistance determinants and antimicrobial susceptibility for all isolates, as well as WGS analysis of five nonenterococcal isolates, can be found in the supplemental material.
