# Supplementary material for: An Optimized Screening Approach for the Oxazolidinone Resistance Gene optrA Yielded a Higher Fecal Carriage Rate among Healthy Individuals in Hangzhou, China
Source: Microbiol Spectr. 2022 Nov 15;10(6):e02974-22. doi: 10.1128/spectrum.02974-22 (PMC9769644; doi:10.1128/spectrum.02974-22)
Supplement: Supplemental file 1 — Tables S1 and S2. Download spectrum.02974-22-s0001.pdf, PDF file, 0.4 MB [file spectrum.02974-22-s0001.pdf]

Supplementary Material

Supplementary Table S1

Carriage of oxazolidinone resistance determinants, and antimicrobial susceptibility for all isolates.

Strain	Species	PCR assays			MIC (mg/L)						
		optrA	*cfr*	*poxtA*	C	LZD	CIP	P	E	VA	TE
A25	*Enterococcus raffinosus*	+	-	-	32	8	>32	>32	32	1	>32
A27	*Ligilactobacillus salivarius*	-	-	+	8	1	≤0.5	≤1	≤0.25	IR	>32
A41	*Enterococcus faecalis*	+	-	-	32	8	>32	2	>32	1	>32
A48	*Enterococcus faecalis*	+	-	-	32	8	>32	2	>32	1	>32
A51	*Enterococcus gallinarum*	+	-	-	64	2	2	2	>32	IR	>32
A53	*Enterococcus avium*	+	-	-	32	8	≤0.5	≤1	≤0.25	1	>32
A65	*Streptococcus gallolyticus*	+	-	-	16	2	1	≤1	>32	0.5	>32
A99	*Enterococcus faecium*	+	-	-	64	8	>32	>32	>32	0.5	>32
A117	*Enterococcus faecium*	+	-	-	32	8	2	16	1	1	≤1
A121	*Enterococcus faecalis*	+	-	-	32	4	2	4	>32	2	>32
A153	*Enterococcus faecalis*	+	-	-	32	4	>32	4	>32	2	>32
A156	*Enterococcus faecium*	-	-	-	32	2	1	4	>32	1	>32
A157	*Enterococcus faecalis*	+	-	-	128	8	>32	4	>32	1	>32
A162	*Enterococcus faecium*	+	-	-	64	8	16	>32	32	1	>32
A167	*Enterococcus faecium*	+	-	-	32	4	2	>32	>32	1	>32
A217	*Enterococcus faecalis*	+	-	-	128	2	1	2	>32	1	>32
A226	*Enterococcus asini*	+	-	-	32	8	1	≤1	4	1	≤1
A245	*Enterococcus hirae*	+	-	-	32	8	≤0.5	2	>32	0.5	>32
A252	*Enterococcus faecium*	+	-	-	32	4	2	4	>32	1	>32
A358	*Enterococcus faecium*	+	-	-	64	4	2	>32	>32	1	>32

A363	*Enterococcus faecalis*	+	-	-	32	8	≤0.5	2	>32	2	>32
A378	*Enterococcus faecalis*	+	-	-	32	8	1	2	32	2	>32
A391	*Enterococcus devriesei*	+	-	-	32	4	2	≤1	≤0.25	0.5	32
A398	*Enterococcus faecalis*	+	-	-	64	8	1	2	>32	1	>32
A400	*Enterococcus faecalis*	+	-	-	32	8	>32	4	>32	2	>32
A440-1	*Enterococcus gallinarum*	+	-	-	32	2	16	16	>32	IR	>32
A440-2	*Enterococcus casseliflavus*	+	-	-	32	8	1	2	32	IR	>32
A464	*Enterococcus faecalis*	+	-	-	64	2	1	2	16	2	>32
A466	*Enterococcus faecalis*	+	-	-	64	8	>32	2	>32	1	>32
A470	*Enterococcus faecalis*	+	-	-	64	8	2	4	>32	1	>32
A528	*Enterococcus faecalis*	+	-	-	32	8	1	2	32	2	>32
A537	*Enterococcus faecalis*	+	-	-	64	4	2	2	>32	1	>32
A539	*Enterococcus casseliflavus*	+	-	-	32	4	16	2	>32	IR	≤1
A547	*Streptococcus gallolyticus*	+	-	-	16	4	4	≤1	>32	1	>32
A555	*Enterococcus faecium*	+	-	-	64	2	4	8	32	1	>32
A556	*Enterococcus faecalis*	+	-	-	128	8	>32	2	>32	1	>32
A566	*Enterococcus faecium*	+	-	-	64	8	8	>32	16	2	>32
A567	*Enterococcus faecalis*	+	-	-	32	8	2	2	>32	2	>32
A569	*Enterococcus casseliflavus*	+	-	-	32	4	4	≤1	32	IR	>32
A592-1	*Enterococcus gallinarum*	+	-	-	64	2	>32	16	32	IR	>32
A592-2	*Enterococcus avium*	+	-	-	16	2	1	≤1	>32	0.5	>32
A605	*Enterococcus faecalis*	+	-	-	32	4	16	2	>32	2	>32
A632	*Enterococcus faecalis*	+	-	-	32	4	2	2	>32	1	>32
A664	*Enterococcus faecalis*	+	-	-	32	8	>32	2	>32	2	>32
A668	*Enterococcus faecium*	+	-	-	32	8	8	>32	>32	1	>32

A669	*Enterococcus faecium*	+	-	-	32	4	2	>32	1	1	>32
A670	*Enterococcus hirae*	-	-	+	8	2	≤0.5	≤1	≤0.25	1	>32
A676	*Enterococcus faecium*	+	-	-	32	4	4	>32	1	1	>32
A677	*Enterococcus avium*	+	-	-	32	4	2	≤1	≤0.25	0.5	≤1
A681	*Enterococcus faecalis*	+	-	-	32	8	1	2	>32	1	>32
A695	*Enterococcus avium*	+	-	-	64	2	1	2	2	1	>32
A753	*Enterococcus faecalis*	+	-	-	128	8	2	4	>32	1	>32
A763	*Enterococcus faecium*	+	-	-	32	4	4	>32	>32	1	>32
A821	*Enterococcus avium*	+	-	-	64	2	≤0.5	2	1	0.5	≤1
A822	*Enterococcus faecium*	+	-	-	32	8	2	4	32	1	>32
A830	*Enterococcus faecalis*	+	-	-	32	8	>32	2	>32	1	>32
A855	*Enterococcus gallinarum*	+	-	-	32	4	1	2	>32	IR	>32
A858	*Enterococcus gallinarum*	+	-	-	32	2	2	4	2	IR	>32
A894	*Enterococcus faecalis*	+	-	-	32	8	2	2	>32	2	>32
A946	*Enterococcus faecalis*	+	-	-	64	8	>32	2	>32	1	>32
A948	*Enterococcus avium*	+	-	-	32	2	1	≤1	1	0.5	>32
A950	*Enterococcus faecalis*	+	-	-	32	8	32	2	>32	1	>32
A964	*Enterococcus faecalis*	+	-	-	32	4	16	2	>32	1	>32
A974	*Lactoococcus garvieae*	+	-	-	32	4	>32	≤1	1	1	≤1
A1010-1	*Enterococcus faecium*	+	-	+	64	4	>32	>32	>32	0.5	>32
A1010-2	*Enterococcus faecium*	+	-	-	64	2	>32	>32	>32	0.5	>32
B3-1	*Enterococcus faecalis*	+	-	-	32	8	1	2	>32	1	>32
B3-2	*Enterococcus devriesei*	+	-	-	32	2	≤0.5	≤1	≤0.25	0.5	>32
B25	*Enterococcus raffinosus*	+	-	-	32	4	>32	>32	32	0.5	>32
B27-1	*Enterococcus thailandicus*	+	-	-	32	4	1	2	4	0.5	>32

B27-2	*Ligilactobacillus salivarius*	-	-	+	8	1	≤0.5	≤1	≤0.25	IR	>32
B33	*Enterococcus faecalis*	+	-	-	32	8	>32	2	>32	2	>32
B36	*Enterococcus faecalis*	+	-	-	32	4	1	2	>32	1	>32
B38	*Enterococcus faecium*	-	-	+	16	4	>32	>32	1	1	>32
B41	*Enterococcus faecalis*	+	-	-	32	8	32	2	>32	1	>32
B47	*Enterococcus faecium*	+	-	-	32	8	2	2	>32	1	≤1
B48	*Enterococcus faecalis*	+	-	-	32	8	>32	2	>32	1	>32
B50	*Enterococcus faecium*	+	-	-	32	8	2	4	>32	2	≤1
B51	*Enterococcus gallinarum*	+	-	-	64	2	2	2	>32	IR	>32
B53	*Enterococcus avium*	+	-	-	32	8	1	≤1	≤0.25	1	>32
B55	*Enterococcus avium*	+	-	-	32	8	1	≤1	≤0.25	0.5	>32
B57	*Enterococcus faecium*	+	-	-	32	8	1	2	>32	1	≤1
B62	*Enterococcus faecalis*	+	-	-	32	8	32	2	>32	2	>32
B65	*Streptococcus gallolyticus*	+	-	-	16	2	1	≤1	>32	0.5	>32
B66	*Enterococcus faecalis*	+	-	-	32	8	16	2	>32	1	>32
B68	*Enterococcus hirae*	+	-	-	32	16	1	≤1	≤0.25	0.5	>32
B72	*Enterococcus avium*	+	-	-	32	16	1	≤1	≤0.25	0.5	>32
B73	*Enterococcus hirae*	+	-	-	32	4	1	2	≤0.25	1	>32
B96	*Enterococcus faecium*	+	-	-	32	8	1	2	≤0.25	1	≤1
B97	*Enterococcus hirae*	+	-	-	64	8	2	2	4	1	≤1
B99	*Enterococcus faecium*	+	-	-	64	16	>32	>32	>32	0.5	>32
B101	*Enterococcus devriesei*	-	-	+	16	2	1	≤1	≤0.25	0.5	16
B113	*Enterococcus faecium*	-	-	-	32	4	1	8	16	1	>32
B117	*Enterococcus faecium*	+	-	-	32	16	4	32	2	1	≤1
B120	*Enterococcus hirae*	+	-	-	32	8	4	2	1	1	>32

B121-1	*Enterococcus faecalis*	+	-	-	32	4	2	4	>32	2	>32
B121-2	*Enterococcus faecalis*	+	-	-	32	4	2	2	8	2	≤1
B127	*Enterococcus faecalis*	+	-	-	64	8	>32	4	>32	1	>32
B129	*Enterococcus faecium*	-	-	+	16	4	4	8	2	1	>32
B133	*Enterococcus faecium*	+	-	+	32	4	8	>32	>32	1	>32
B141	*Enterococcus faecalis*	+	-	-	32	4	1	2	>32	1	>32
B149	*Enterococcus faecalis*	+	-	-	32	8	>32	2	>32	1	>32
B153	*Enterococcus faecalis*	+	-	-	32	8	>32	2	>32	1	>32
B156	*Enterococcus faecium*	-	-	-	32	4	1	8	>32	2	>32
B157	*Enterococcus faecalis*	+	-	-	128	8	>32	4	>32	2	>32
B160-1	*Enterococcus durans*	+	-	-	64	4	32	16	16	1	>32
B160-2	*Enterococcus avium*	+	-	-	32	4	1	≤1	>32	0.5	>32
B162-1	*Enterococcus faecium*	+	-	-	64	16	16	>32	16	1	>32
B162-2	*Enterococcus faecium*	+	-	-	32	16	2	2	>32	1	>32
B166	*Enterococcus faecalis*	+	-	-	64	8	1	4	>32	2	>32
B167	*Enterococcus faecalis*	+	-	-	32	8	32	2	>32	1	>32
B169	*Enterococcus casseliflavus*	+	-	-	32	4	1	≤1	>32	IR	>32
B173	*Enterococcus faecium*	+	-	-	64	8	16	32	>32	1	>32
B178-1	*Enterococcus faecalis*	+	-	-	32	8	1	2	>32	2	>32
B178-2	*Enterococcus faecium*	-	-	+	16	4	1	4	>32	2	>32
B183	*Enterococcus faecalis*	+	-	-	128	8	32	2	>32	1	>32
B196-1	*Enterococcus faecalis*	+	-	-	64	8	2	2	32	1	>32
B196-2	*Enterococcus faecium*	-	-	+	16	4	1	8	>32	1	>32
B214	*Enterococcus faecium*	+	-	-	64	4	4	>32	>32	0.5	>32
B217	*Enterococcus faecalis*	+	-	-	128	2	1	2	>32	1	>32

B226-1	*Enterococcus gallinarum*	+	-	-	64	8	>32	32	>32	IR	>32
B226-2	*Enterococcus asini*	+	-	-	32	8	2	≤1	4	0.5	≤1
B244	*Enterococcus faecium*	+	-	-	32	8	4	4	4	1	≤1
B245-1	*Enterococcus faecalis*	+	-	-	32	16	>32	2	>32	1	>32
B245-2	*Enterococcus cecorum*	+	-	-	16	4	>32	≤1	>32	0.5	>32
B248	*Enterococcus faecium*	+	-	+	64	4	4	2	16	2	>32
B252-1	*Enterococcus faecium*	+	-	+	64	4	4	>32	>32	1	>32
B252-2	*Enterococcus faecium*	+	-	-	64	16	8	>32	2	2	≤1
B264	*Enterococcus faecalis*	+	-	-	64	8	1	2	>32	1	>32
B274	*Enterococcus faecalis*	+	-	-	64	8	>32	2	>32	1	≤1
B276	*Enterococcus hirae*	+	-	-	32	16	1	2	8	1	>32
B280	*Enterococcus avium*	+	-	-	16	4	1	2	>32	0.5	>32
B288	*Enterococcus faecalis*	+	-	-	32	8	≤0.5	2	>32	1	>32
B303-1	*Enterococcus faecalis*	+	-	-	32	8	1	2	>32	2	>32
B303-2	*Enterococcus faecium*	+	-	-	32	16	4	>32	16	1	>32
B303-3	*Enterococcus faecium*	+	-	-	32	16	8	>32	>32	1	>32
B316	*Enterococcus casseliflavus*	+	-	-	128	8	2	2	>32	IR	>32
B343	*Enterococcus faecalis*	+	-	-	32	4	1	2	2	2	>32
B347	*Enterococcus faecalis*	+	-	-	32	8	>32	2	>32	1	>32
B353	*Enterococcus faecalis*	+	-	-	64	8	2	2	16	1	>32
B358	*Enterococcus faecium*	+	-	-	64	4	4	>32	>32	1	>32
B359	*Enterococcus faecalis*	+	-	-	32	4	1	2	>32	2	>32
B361-1	*Enterococcus faecium*	+	-	-	64	16	16	>32	>32	0.5	>32
B361-2	*Enterococcus faecium*	+	-	+	32	4	8	>32	>32	1	>32
B361-3	*Enterococcus hirae*	+	-	-	64	8	1	2	4	1	>32

B363	*Enterococcus faecalis*	+	-	-	32	8	1	2	>32	1	>32
B366	*Enterococcus faecalis*	+	-	-	128	8	1	4	>32	2	>32
B369	*Enterococcus hirae*	-	-	+	16	4	4	2	≤0.25	1	>32
B378-1	*Enterococcus faecalis*	+	-	-	32	16	2	2	>32	2	>32
B378-2	*Enterococcus faecium*	-	-	+	16	8	1	8	16	2	>32
B389	*Enterococcus faecium*	-	-	+	16	4	16	8	>32	2	>32
B391-1	*Enterococcus hirae*	+	-	-	32	16	1	2	≤0.25	0.5	>32
B391-2	*Vagococcus lutrae*	+	-	-	32	8	1	2	≤0.25	0.5	>32
B391-3	*Enterococcus dongliensis*	-	-	+	16	2	4	≤1	>32	2	32
B398-1	*Enterococcus faecalis*	+	-	-	64	8	32	2	>32	2	>32
B398-2	*Enterococcus hirae*	+	-	-	32	16	4	≤1	>32	1	>32
B399	*Enterococcus faecalis*	+	-	-	64	8	2	2	32	2	>32
B400	*Enterococcus faecalis*	+	-	-	32	8	>32	4	>32	2	>32
B405	*Enterococcus avium*	+	-	-	64	2	>32	16	>32	1	≤1
B411	*Enterococcus casseliflavus*	+	-	-	32	16	2	≤1	32	IR	≤1
B412-1	*Enterococcus faecalis*	+	-	-	32	8	2	4	32	1	>32
B412-2	*Enterococcus faecalis*	+	-	-	32	8	1	4	>32	1	>32
B413	*Enterococcus avium*	+	-	-	32	4	1	≤1	32	1	>32
B415	*Enterococcus faecalis*	+	-	-	64	2	2	2	16	1	>32
B417	*Enterococcus casseliflavus*	+	-	-	64	16	32	2	>32	IR	>32
B419	*Enterococcus casseliflavus*	+	-	-	64	16	32	2	>32	IR	>32
B423-1	*Enterococcus gallinarum*	+	-	-	32	4	8	>32	>32	IR	>32
B423-2	*Enterococcus casseliflavus*	+	-	-	32	8	1	2	>32	IR	>32
B424-1	*Enterococcus gallinarum*	+	-	-	32	4	8	>32	>32	IR	>32
B424-2	*Enterococcus casseliflavus*	+	-	-	32	8	1	≤1	>32	IR	>32

B425-1	*Enterococcus gallinarum*	+	-	-	32	4	8	>32	>32	IR	>32
B425-2	*Enterococcus casseliflavus*	+	-	-	32	8	1	≤1	>32	IR	>32
B440-1	*Enterococcus faecalis*	+	-	-	32	8	>32	2	>32	1	>32
B440-2	*Enterococcus gallinarum*	+	-	-	32	4	8	>32	>32	IR	>32
B440-3	*Enterococcus casseliflavus*	+	-	-	32	8	1	≤1	>32	IR	>32
B448	*Enterococcus faecalis*	+	-	-	32	8	2	2	>32	2	>32
B464	*Enterococcus faecalis*	+	-	-	32	2	1	2	8	1	>32
B466	*Enterococcus faecalis*	+	-	-	64	8	>32	4	>32	1	>32
B467	*Enterococcus faecalis*	+	-	-	64	8	1	2	>32	1	>32
B470	*Enterococcus faecalis*	+	-	-	64	8	2	2	32	1	>32
B471-1	*Enterococcus gallinarum*	+	-	-	64	16	1	2	>32	IR	>32
B471-2	*Enterococcus casseliflavus*	+	-	-	64	16	2	2	8	IR	>32
B471-3	*Enterococcus avium*	+	-	-	32	4	≤0.5	≤1	>32	0.5	>32
B475	*Enterococcus faecalis*	+	-	-	64	8	>32	2	>32	1	>32
B477	*Enterococcus avium*	+	-	-	32	4	≤0.5	2	>32	1	≤1
B479	*Enterococcus faecalis*	+	-	-	32	8	≤0.5	2	8	1	>32
B480	*Enterococcus faecalis*	+	-	-	64	8	2	2	>32	2	>32
B492	*Enterococcus faecalis*	+	-	-	64	8	>32	2	>32	1	>32
B494	*Enterococcus faecium*	+	-	-	32	4	>32	>32	>32	0.5	>32
B495	*Enterococcus raffinosus*	+	+	-	128	2	≤0.5	>32	1	0.5	>32
B501	*Enterococcus faecalis*	+	-	-	32	8	1	2	>32	1	>32
B502	*Enterococcus casseliflavus*	+	-	-	64	4	8	≤1	>32	IR	>32
B510	*Enterococcus hirae*	-	-	+	16	4	1	2	≤0.25	1	>32
B511	*Enterococcus hirae*	-	-	+	16	4	1	8	>32	0.5	>32
B512	*Enterococcus faecium*	+	-	-	64	16	4	>32	>32	1	>32

B513	*Enterococcus faecium*	+	-	-	32	8	2	>32	1	1	>32
B516	*Enterococcus casseliflavus*	+	+	-	32	4	32	2	>32	IR	>32
B521	*Enterococcus faecalis*	+	-	-	128	8	>32	2	>32	1	>32
B528	*Enterococcus faecalis*	+	-	-	32	8	1	2	>32	1	>32
B530	*Enterococcus faecalis*	+	-	-	64	8	>32	2	>32	1	>32
B537	*Enterococcus faecalis*	+	-	-	64	8	2	2	>32	1	>32
B539	*Enterococcus casseliflavus*	+	-	-	64	16	1	2	>32	IR	>32
B545	*Enterococcus faecium*	+	-	-	32	16	4	4	16	2	>32
B546	*Enterococcus faecalis*	+	-	-	32	4	2	4	>32	2	>32
B553	*Enterococcus faecium*	+	-	-	64	4	4	8	32	1	>32
B554	*Enterococcus casseliflavus*	+	-	-	32	2	1	2	>32	IR	>32
B555-1	*Enterococcus faecium*	+	-	-	32	16	8	>32	>32	1	>32
B555-2	*Enterococcus faecium*	+	-	-	64	4	4	4	>32	1	>32
B556	*Enterococcus faecalis*	+	-	-	64	8	2	2	>32	2	>32
B560	*Enterococcus faecium*	+	-	-	64	4	4	8	>32	1	>32
B562	*Enterococcus faecalis*	+	-	-	64	8	2	2	>32	1	≤1
B563	*Enterococcus faecalis*	+	-	-	32	8	1	2	>32	1	>32
B566	*Enterococcus faecium*	+	-	-	64	8	4	>32	32	1	>32
B567	*Enterococcus faecalis*	+	-	-	32	8	2	2	>32	1	>32
B569	*Enterococcus casseliflavus*	+	-	-	32	4	4	2	32	IR	>32
B573	*Enterococcus faecalis*	+	-	-	64	8	1	2	>32	1	>32
B575	*Enterococcus thailandicus*	+	-	-	64	2	1	>32	>32	1	>32
B579	*Enterococcus faecalis*	+	-	-	64	8	>32	2	>32	1	>32
B582	*Enterococcus thailandicus*	+	-	-	32	4	1	≤1	>32	0.5	>32
B583-1	*Enterococcus faecalis*	+	-	-	64	8	2	2	32	1	>32

B583-2	*Enterococcus faecalis*	+	-	-	64	8	32	2	>32	1	>32
B592-1	*Enterococcus faecalis*	+	-	-	32	8	>32	2	>32	1	>32
B592-2	*Enterococcus gallinarum*	+	-	-	128	4	>32	32	32	IR	>32
B599-1	*Enterococcus faecalis*	+	-	-	64	8	1	2	>32	1	>32
B599-2	*Enterococcus faecalis*	+	-	-	32	8	4	4	>32	1	>32
B604	*Enterococcus casseliflavus*	+	-	-	32	4	2	2	8	IR	>32
B605	*Enterococcus faecalis*	+	-	-	32	8	32	2	>32	1	>32
B608	*Enterococcus casseliflavus*	+	-	-	64	2	1	2	>32	IR	>32
B627	*Enterococcus faecium*	-	-	+	32	8	4	>32	2	1	>32
B628	*Enterococcus faecium*	+	-	-	32	16	>32	>32	>32	1	>32
B632	*Enterococcus faecalis*	+	-	-	64	8	2	2	>32	1	>32
B646-1	*Enterococcus faecalis*	+	-	-	64	8	>32	2	>32	1	>32
B646-2	*Enterococcus raffinosus*	-	-	+	32	4	≤0.5	>32	>32	1	>32
B655	*Enterococcus faecalis*	+	-	-	64	8	1	4	>32	2	>32
B656-1	*Enterococcus faecalis*	+	-	-	64	8	1	4	>32	1	>32
B656-2	*Enterococcus faecalis*	+	-	-	32	8	>32	≤1	>32	1	>32
B663-1	*Enterococcus faecium*	+	-	-	>128	8	4	>32	>32	1	>32
B663-2	*Enterococcus faecium*	+	-	-	64	8	2	>32	>32	0.5	>32
B664	*Enterococcus faecalis*	+	-	-	32	8	>32	2	>32	1	>32
B668	*Enterococcus faecium*	+	-	-	64	16	8	>32	>32	1	>32
B669-1	*Enterococcus faecium*	+	-	-	32	8	4	>32	1	1	>32
B669-2	*Enterococcus thailandicus*	+	-	-	32	4	1	2	2	1	>32
B676	*Enterococcus faecium*	+	-	-	32	8	4	>32	0.5	1	>32
B677	*Enterococcus avium*	+	-	-	32	4	2	2	≤0.25	0.5	≤1
B681	*Enterococcus faecalis*	+	-	-	32	8	1	4	>32	1	>32

B686	*Enterococcus faecium*	+	-	-	32	4	8	>32	>32	0.5	>32
B687-1	*Enterococcus faecium*	+	-	-	64	16	2	>32	>32	2	>32
B687-2	*Enterococcus faecium*	+	-	-	32	16	2	>32	32	1	>32
B693	*Enterococcus faecalis*	+	-	-	32	8	1	2	>32	1	>32
B695	*Enterococcus avium*	+	-	-	64	2	≤0.5	2	1	0.5	>32
B700	*Enterococcus faecalis*	+	-	-	32	8	>32	2	>32	1	>32
B706	*Enterococcus faecalis*	+	-	-	128	8	1	2	>32	1	>32
B717	*Enterococcus faecalis*	+	-	-	128	8	1	4	>32	1	>32
B732	*Enterococcus avium*	+	+	-	64	4	1	>32	>32	0.5	>32
B742	*Enterococcus faecalis*	+	-	-	32	8	>32	2	>32	1	>32
B745	*Enterococcus avium*	+	-	-	64	4	1	2	>32	1	>32
B746-1	*Enterococcus faecalis*	+	-	-	64	8	1	4	>32	1	>32
B746-2	*Enterococcus faecium*	+	-	-	64	4	4	>32	>32	1	>32
B749	*Enterococcus faecium*	+	-	+	32	8	>32	>32	>32	0.5	>32
B753	*Enterococcus faecalis*	+	-	-	128	8	1	2	>32	1	>32
B759	*Enterococcus faecium*	+	-	-	64	16	4	>32	16	0.5	>32
B762	*Enterococcus faecalis*	+	-	-	32	4	1	2	>32	1	>32
B763	*Enterococcus faecium*	+	-	-	32	4	4	>32	>32	1	>32
B766	*Enterococcus faecalis*	+	-	-	32	8	>32	2	>32	1	>32
B778-1	*Enterococcus faecalis*	+	-	-	32	8	1	4	>32	1	>32
B778-2	*Enterococcus faecium*	+	-	-	32	4	8	>32	>32	1	>32
B785-1	*Enterococcus faecium*	+	-	-	32	16	2	8	1	1	>32
B785-2	*Enterococcus avium*	+	-	-	64	2	≤0.5	2	0.5	0.5	>32
B788	*Enterococcus hirae*	+	-	-	64	8	1	2	32	1	>32
B802	*Enterococcus faecalis*	+	-	-	64	4	2	2	>32	1	>32

B806	*Enterococcus faecalis*	+	-	-	128	8	2	2	>32	2	>32
B821	*Enterococcus avium*	+	-	-	64	4	≤0.5	≤1	1	0.5	≤1
B822	*Enterococcus faecium*	+	-	-	64	8	2	2	>32	1	>32
B827	*Lactococcus formosensis*	+	-	-	64	8	2	≤1	>32	0.5	>32
B829	*Enterococcus faecalis*	+	-	-	32	8	2	2	32	1	>32
B830	*Enterococcus faecalis*	+	-	-	32	16	32	2	>32	1	>32
B834	*Enterococcus avium*	+	-	-	64	2	1	2	1	1	>32
B841	*Enterococcus gallinarum*	+	-	-	32	4	1	2	1	IR	>32
B843	*Enterococcus gallinarum*	+	-	-	32	4	2	4	4	IR	>32
B851	*Enterococcus faecalis*	+	-	-	32	16	1	2	1	1	>32
B855	*Enterococcus gallinarum*	+	-	-	32	4	1	2	>32	IR	>32
B858-1	*Enterococcus gallinarum*	+	-	-	64	4	>32	32	>32	IR	>32
B858-2	*Enterococcus gallinarum*	+	-	-	32	2	2	4	1	IR	>32
B882	*Enterococcus faecalis*	+	-	-	32	4	2	2	>32	1	>32
B892	*Enterococcus faecalis*	+	-	-	32	16	>32	2	>32	1	>32
B894	*Enterococcus faecalis*	+	-	-	32	8	2	2	>32	1	>32
B898	*Enterococcus faecalis*	+	-	-	16	2	1	2	1	1	>32
B899	*Enterococcus gallinarum*	+	-	-	32	4	>32	16	>32	IR	≤1
B904	*Enterococcus thailandicus*	+	-	+	32	4	>32	4	32	1	>32
B916	*Enterococcus faecalis*	+	-	-	128	4	32	2	>32	1	>32
B918	*Enterococcus faecalis*	+	-	-	128	2	2	2	>32	1	>32
B946	*Enterococcus faecalis*	+	-	-	64	8	>32	2	>32	1	>32
B948	*Enterococcus avium*	+	-	-	64	4	1	≤1	1	0.5	>32
B950	*Enterococcus faecalis*	+	-	-	32	8	>32	2	>32	1	>32
B951	*Enterococcus faecalis*	+	-	-	32	8	32	2	>32	1	>32

B952	*Enterococcus faecalis*	+	-	-	128	8	1	2	>32	1	>32
B953	*Enterococcus avium*	+	-	-	32	8	1	32	>32	0.5	>32
B958	*Enterococcus faecalis*	+	-	-	64	8	1	2	>32	1	>32
B959	*Enterococcus faecalis*	+	-	-	128	8	32	2	>32	1	>32
B962	*Enterococcus faecium*	+	-	-	32	16	4	4	0.5	1	>32
B964	*Enterococcus faecalis*	+	-	-	32	4	32	2	>32	1	>32
B973	*Enterococcus faecium*	+	-	-	32	16	4	2	8	1	>32
B974	*Lactoococcus garvieae*	+	-	-	32	4	>32	2	≤0.25	1	≤1
B979	*Enterococcus faecalis*	+	-	-	64	8	1	2	32	1	>32
B988	*Enterococcus hirae*	+	-	-	32	2	1	≤1	>32	1	>32
B990	*Enterococcus faecalis*	+	-	-	128	8	>32	4	>32	1	>32
B991	*Enterococcus faecalis*	+	-	-	128	8	1	2	>32	1	>32
B1000-1	*Enterococcus faecalis*	+	-	+	64	8	1	2	>32	1	>32
B1000-2	*Enterococcus faecalis*	+	-	-	128	4	2	2	>32	1	>32
B1006	*Enterococcus faecalis*	+	-	-	64	8	1	2	>32	1	>32
B1009	*Enterococcus faecium*	+	-	+	64	4	>32	>32	>32	0.5	>32
B1010-1	*Enterococcus faecalis*	+	-	-	32	8	>32	2	>32	2	>32
B1010-2	*Enterococcus faecium*	+	-	+	64	8	>32	>32	>32	0.5	>32

C, chloramphenicol; LZD, linezolid; CIP, ciprofloxacin; P, penicillin G; E, erythromycin; VA, vancomycin; TE, tetracycline; IR, intrinsic resistant.

Supplementary Table S2

OptrA variants and carriage of antimicrobial-resistance determinants for six nonenterococcal isolates

**Isolate**	**OptrA variants**	**Carriage of antimicrobial-resistance determinants**
Streptococcus gallolyticus A547	IDKKGPM	*optrA, fexA, erm*(A)*, erm*(B)*, lnu*(B)*, lsa*(E)*, tet*(L)*, tet*(O)*, tet*(O/W/32/O)*, ant(6)-Ia*
Streptococcus gallolyticus B65	IDKKGP	*optrA, fexA, erm*(B)*, tet*(M)
Vagococcus lutrae B391-2	KLDK	*optrA, fexA, lnu*(G)*, tet*(M)
Lactococcus formosensis B827	Wild type	*optrA, fexA, erm*(B)*, lnu*(B)*, lsa*(E)*, tet*(L)*, tet*(O/W/32/O)*, tet*(S)*, aac(6')-aph(2''), ant(6)-Ia, mdt*(A)
Lactococcus garvieae B974	EYDDI	*optrA, fexA, mdt*(A)
Ligilactobacillus salivarius B27-2	NA	*poxtA, fexB, lnu*(A)*, tet*(L)*, tet*(M)
